# A Hierarchically Ordered Mesoporous-Carbon-Supported Iron Sulfide Anode for High-Rate Na-Ion Storage

**DOI:** 10.3390/molecules26144349

**Published:** 2021-07-18

**Authors:** Anupriya K. Haridas, Natarajan Angulakshmi, Arul Manuel Stephan, Younki Lee, Jou-Hyeon Ahn

**Affiliations:** 1 Department of Materials Engineering and Convergence Technology, Gyeongsang National University, 501 Jinju-daero, Jinju 52828, Korea; anupriya.haridas@gmail.com (A.K.H.); anguluxmi@gmail.com (N.A.); 2 Electrochemical Power Sources Division, CSIR—Central Electrochemical Research Institute, Karaikudi 630 006, India; arulmanuel@gmail.com

**Keywords:** Iron sulfide, ordered mesoporous carbon, high-rate anode, sodium-ion battery

## Abstract

Sodium-ion batteries (SIBs) are promising alternatives to lithium-based energy storage devices for large-scale applications, but conventional lithium-ion battery anode materials do not provide adequate reversible Na-ion storage. In contrast, conversion-based transition metal sulfides have high theoretical capacities and are suitable anode materials for SIBs. Iron sulfide (FeS) is environmentally benign and inexpensive but suffers from low conductivity and sluggish Na-ion diffusion kinetics. In addition, significant volume changes during the sodiation of FeS destroy the electrode structure and shorten the cycle life. Herein, we report the rational design of the FeS/carbon composite, specifically FeS encapsulated within a hierarchically ordered mesoporous carbon prepared via nanocasting using a SBA-15 template with stable cycle life. We evaluated the Na-ion storage properties and found that the parallel 2D mesoporous channels in the resultant FeS/carbon composite enhanced the conductivity, buffered the volume changes, and prevented unwanted side reactions. Further, high-rate Na-ion storage (363.4 mAh g^−1^ after 500 cycles at 2 A g^−1^, 132.5 mAh g^−1^ at 20 A g^−1^) was achieved, better than that of the bare FeS electrode, indicating the benefit of structural confinement for rapid ion transfer, and demonstrating the excellent electrochemical performance of this anode material at high rates.

## 1. Introduction

Although the commercialization of lithium-ion battery (LIB) technology has led to the development of electric vehicles and portable electronic devices, inadequate global lithium resources have limited further advancements in the energy storage sector [1]. In particular, the significant exploitation of lithium resources in recent years has resulted in an increased scarcity of lithium resources and substantial price hikes. Therefore, sodium-ion batteries (SIBs) have gained wide popularity, mainly due to of the global abundance of sodium resources and the striking similarity of the ion-insertion mechanism in SIBs and LIBs. Thus, the natural abundance, and associated low cost, of sodium make SIBs suitable for large-scale energy storage applications [2]. However, conventional LIB anode materials cannot undergo reversible sodiation reactions, resulting in inadequate capacities. Furthermore, both the theoretical and experimental exploration of carbon materials, MXenes, and transition metal dichalcogenides as anode materials for SIBs demonstrate that the search for an ideal SIB anode is an ongoing challenge [3,4,5,6].

Conversion-based transition metal sulfides (TMS, TM = Co, Fe, and Cu) have been explored extensively as anode materials for SIBs, due to their high theoretical capacities and widespread availability [7,8,9]. Among the commonly studied metal sulfides, iron-based sulfides have drawn attention, owing to their natural abundance, cost-effectiveness, and eco-friendliness [10,11,12]. However, the low conductivity and inherently sluggish Na-ion diffusion kinetics arising from the large ionic radius of Na^+^, as well as the generation of polysulfide intermediates, limit the electrochemical performance of these materials [13]. In addition, during the sodiation of the conversion materials, significantly larger volume changes occur compared to the lithiation process, resulting in pronounced strain in the electrode, eventually shortening the cycle life. To overcome these shortcomings, the rational design of the FeS/carbon composites that can deliver high-performance Na-ion storage is necessary. One strategy for the preparation of these composites is the encapsulation within carbon matrices, such as carbon nanotubes (CNTs) and graphene; this strategy can increase the cyclability of the conversion materials by buffering the strain amidst the volume changes and limiting the polysulfide shuttling process that occurs during electrochemical cycling [14,15,16]. To date, various porous and nonporous carbon matrices have been employed as conductive supports to facilitate electrolyte percolation, thus enabling the rapid ion/electron transfer in batteries [17,18]. For example, sophisticated gyroid 3D networks based on silica-based templates have shown excellent volume sustainability for Si anodes in LIBs [19,20].

Ordered mesoporous carbon (OMC) is a carbon matrix class containing hierarchically ordered mesoporous channels that endow 3D pore connectivity. The synthesis of 2D hexagonal OMCs, using ordered mesoporous silica as a template, was reported for the first time in 2000 by Jun et al., and subsequent research revealed many new applications of these mesoporous carbon materials [21]. Since then, OMCs with various mesopore structures, such as the so-called CMK-n (carbon mesostructured by KAIST) series, have been prepared via nanocasting, utilizing mesoporous silica or aluminosilicate hard templates [22]. OMCs form an ideal conductive carbon host, having a high surface area and interconnected mesoporous channels, enabling active material confinement and sufficient electrolyte percolation for redox reactions. The successful confined growth of a sulfur species within a hierarchically porous OMC structure with parallel channels was reported by Nazar et al. in 2009, and exhibited encouraging electrochemical properties [23]. Currently, there are many methods to generate active material/OMC nanocomposites, including nanocasting [24,25], sonochemical synthesis [26,27], and hydrothermal-assisted growth [28,29]. To date, such strategies have provided interesting results, enabling high-rate and stable cycling properties for alkali-ion-based energy storage [30].

In our earlier work, we reported high specific area modified mesoporous carbons as sulfur hosts that can reduce the generation and shuttling of polysulfide species, thereby enabling excellent electrochemical properties in lithium-sulfur batteries using SBA-15 as a template [31,32]. Followingly, in this study, we report the in situ synthesis of FeS within an OMC structure, CMK-3, prepared by nanocasting using an SBA-15 hard template with 2D hexagonal *P*6*mm* symmetry. The in situ generation of FeS was carried out within the OMCs via a simple aqueous synthetic method. During synthesis, the acid-functionalized hierarchically ordered mesoporous OMCs (*f*-OMCs) act as sites for the nucleation of FeS nanoparticles and simultaneously prevents the unwanted aggregation and growth of nanoparticles within the 2D mesoporous during the crystallization process. As FeS electrodes are prone to the pulverization, cracking, and isolation of active materials, it is important to incorporate conductive matrices that can accommodate volume changes, while also being in close contact with the active material and the electrolyte. Although CMK-3 is an open 2D structure, it can sustain the volume change of FeS and has fast ion transport properties, due to its good contact area with the nanosized FeS. In addition, the mesoporous carbon framework acts as a conductive reservoir and effectively prevents the shuttling of polysulfides. Thus, the employed structural design, with an open carbon framework and high surface area, enhances the electrolyte percolation pathways and buffers the volume changes effectively. The systematic evaluation of the Na-ion storage properties of the synthesized FeS incorporated OMCs (FeS@*f*-OMC) revealed a stable discharge capacity of 363 mAh g^−1^ after 500 cycles at 2 A g^−1^ and exhibited excellent high-rate Na-ion storage (132.5 mAh g^−1^ at 20 A g^−1^), owing to the hierarchical mesoporous structure of the carbon host that supports the FeS active material.

## 2. Results

### 2.1. Synthesis and Characterization of the FeS@f-OMC Composite

**Figure 1:**
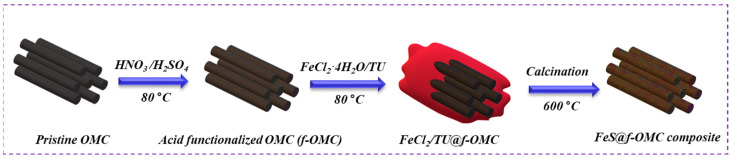
Schematic of the synthesis of the FeS@*f*-OMC composite.

Figure 1 shows a schematic of the synthesis of the FeS@*f*-OMC composite. A simple aqueous chemical route was chosen for the synthesis of FeS embedded OMC, utilizing iron chloride, thiourea, and acid-treated *f*-OMCs as the iron source, sulfur source, and carbon framework, respectively. Initially, the OMC was acid-functionalized to improve the hydrophilicity and facilitate dispersion. After that, the prepared *f*-OMC was dispersed in 1:1 (*v*/*v*) deionized (DI) water and ethanol, and the iron and sulfur precursors were added sequentially, followed by thorough stirring. During this process, the dissolved iron and sulfur species infiltrate the 2D mesoporous channels via the capillary effect, resulting in the slow nucleation and subsequent growth of the FeS nanoparticles within, as well as outside of, the *f*-OMC structure. Final high-temperature heat treatment resulted in the formation of a black powder, the FeS-embedded *f*-OMC composite (i.e., FeS@*f*-OMC). Importantly, the gradual growth of the FeS nanoparticles within the mesoporous channels helped to control the nucleation and growth of FeS particles within, and on, the framework of the mesoporous channels, preventing agglomeration.

**Figure 2:**
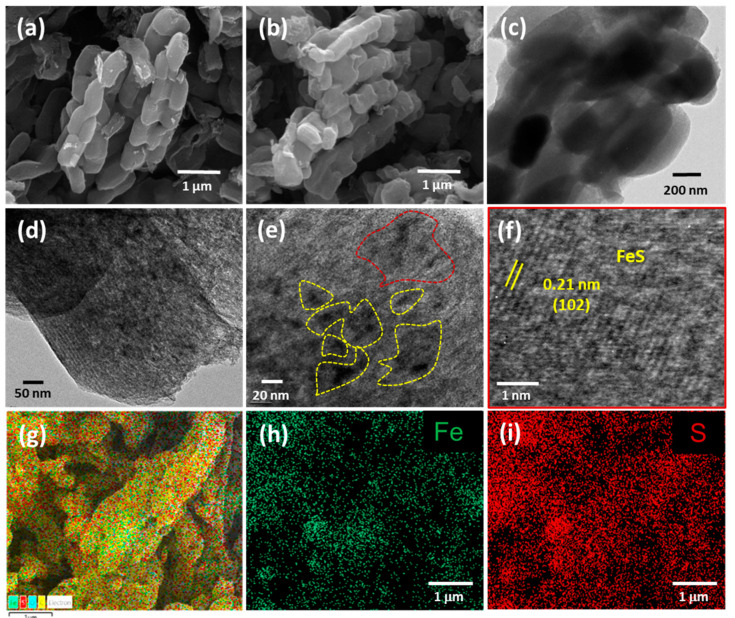
FE-SEM images of (**a**) *f*-OMC and (**b**) FeS@*f*-OMC. TEM images (**c**,**d**) of FeS@*f*-OMC. HR-TEM images (**e**,**f**) and EDS (**g**) map overlaid over a high-angle annular dark-field image of FeS@*f*-OMC and individual EDS maps of iron (**h**) and sulfur (**i**) in the composite.

The morphology of both the pristine *f*-OMC and the FeS@*f*-OMC composites was analyzed using field-emission scanning electron microscopy (FE-SEM), as shown in Figure 2a,b. Both samples exhibited similar morphologies, implying that the structure of the *f*-OMC was preserved after the nucleation and growth of FeS nanoparticles. The pristine FeS consisted of irregular bulk particles, as shown in Figure S3. Further micro structural analysis of the FeS@*f*-OMC composite, carried out using transmission electron microscopy (TEM), revealed the mesoporous channels of the OMC structure in both the pristine *f*-OMC and the FeS@*f*-OMC composite (Figure 2c,d). However, in the case of the FeS@*f*-OMC composite, clusters of nanoparticles (regions of dark contrast) were also observed along with the mesoporous channels (Figure 2d). Further analysis, using high-resolution (HR)-TEM, revealed the (102) plane of hexagonal FeS, indicating the successful formation of FeS within the OMC. It should be noted that the ordered mesoporous channels of the OMC cannot be seen clearly because of the presence of the FeS nanoparticles. X-ray energy dispersive spectroscopy (EDS) mapping of the composite revealed the uniform and homogeneous distribution of iron and sulfur within the synthesized composite.

**Figure 3:**
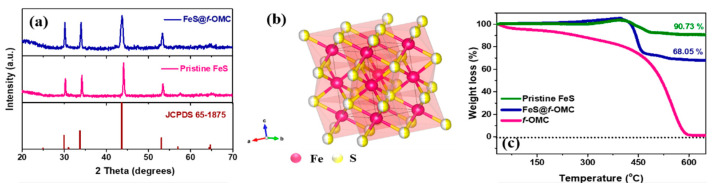
(**a**) XRD patterns of FeS@*f*-OMC, pristine FeS, and pristine OMC, (**b**) structure of FeS, and (**c**) TGA curves obtained in air.

The structural analysis of the FeS@*f*-OMC composite was performed via X-ray diffraction (XRD) (Figure 3). The observed XRD peaks can be assigned to JCPDS card number 65-1875, corresponding to hexagonal FeS. The pristine FeS, synthesized without OMC, showed similar structural characteristics. The thermogravimetric analysis (TGA) of pristine OMC, FeS@*f*-OMC composite, and pristine FeS was performed in air to assess the thermal stability, and to determine the active material and carbon contents in the FeS@*f*-OMC composite. The pristine OMC showed a continuous mass loss at room temperature, unlike the FeS@*f*-OMC composite and pristine FeS samples. The FeS@*f*-OMC composite and pristine FeS samples showed similar trends of mass increase after 210 °C, due to the partial oxidation of FeS to higher-molecular-weight FeSO_4_ [33]. Subsequently, both samples showed a gradual weight loss beyond 400 °C, due to the transformation of FeSO_4_ to Fe_2_O_3_ [33]. The additional weight loss observed in the FeS@*f*-OMC composite sample can be attributed to the simultaneous oxidation of the OMC framework. XRD analysis of the final product after the heat-treatment process was carried out to confirm the formation of Fe_2_O_3_ (Figure S4). By comparing the TGA curves, the contents of FeS and carbon in the FeS@*f*-OMC composite were estimated to be 75% and 15%, respectively (details of these calculations are given in the Supplementary Information (SI)).

**Figure 4:**
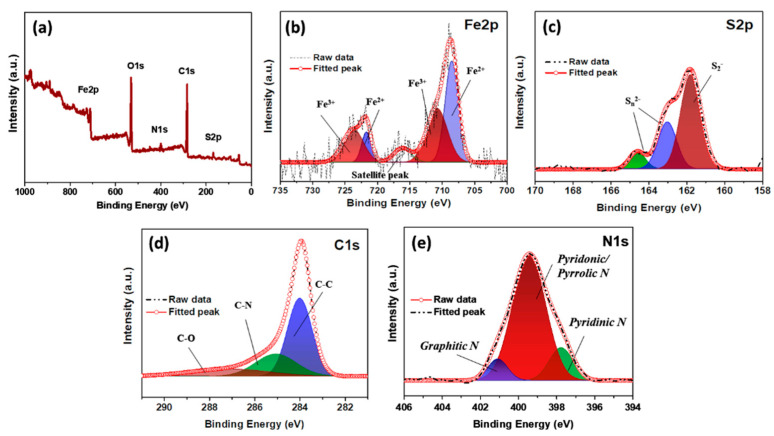
XPS (**a**) survey spectrum and high-resolution, (**b**) Fe 2p, (**c**) S 2p, (**d**) C 1s, and (**e**) N 1s spectra of the FeS@*f*-OMC composite.

Additionally, the chemical states of the iron and sulfur in the composite were analyzed using X-ray photoelectron spectroscopy (XPS) (Figure 4a–e). The two major peaks in the Fe 2p spectrum were deconvoluted into two peaks each, suggesting the presence of Fe^2+^ (708.5 and 721.7 eV) and Fe^3+^ (710.7 and 723.6 eV) [34]. The S 2p spectrum of the composite contains three major peaks located at 161.7, 163.0, and 164.5 eV, which indicates a metal–sulfide bond, the S 2p1/2, and S 2p3/2 states, respectively [33]. The C 1s spectrum contains three major peaks, centered at 284.2, 285.1, and 286.9 eV, indicating C-C, C-N, and C-O (epoxy or alkoxy) bonds, respectively [35]. Additionally, the N 1s spectrum contains peaks that can be attributed to pyridinic (397.7 eV), pyridonic/pyrrolic (399.5 eV), and graphitic (401.2 eV) N species in the composite.

**Figure 5:**
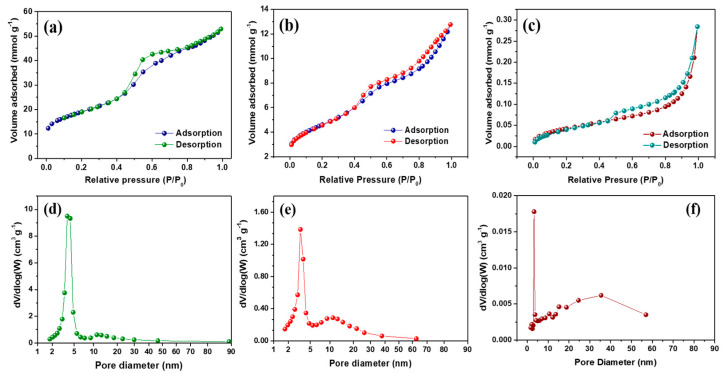
Brunauer–Emmett–Teller adsorption–desorption isotherms of (**a**) *f*-OMC, (**b**) FeS@*f*-OMC, and (**c**) pristine FeS using N_2_ gas and (**d**–**f**) the corresponding pore size distribution curves.

The N_2_ adsorption–desorption isotherms and the Barrett–Joyner–Halenda (BJH) pore size distribution of *f*-OMC and FeS@*f*-OMC composites are presented in Figure 5. The adsorption–desorption isotherms display hysteresis loops in the relative pressure (*P*/*P*_0_) range of 0.4–0.8, indicating a type-H_2_ isotherm, which indicates the presence of mesopores, according to the IUPAC classification. The *f*-OMC sample was found to have a high specific surface area of 1505 m^2^ g^−1^, whereas that of the FeS@*f*-OMC composite was lower (364 m^2^ g^−1^). The low specific surface area observed for FeS@*f*-OMC can be attributed to the partial filling of the mesopores by the in situ formation of FeS nanoparticles during synthesis. This change is also manifested in the pore size distribution curves of the FeS@*f*-OMC composite and *f*-OMCs. Notably, pristine OMC possesses prominent mesopores within the ranges of 3.7–4.2 and 10–20 nm. However, the incorporation of FeS nanoparticles resulted in the partial filling of the mesopores, and the number of mesopores in the indicated range was reduced considerably. At the same time, the pristine FeS particles were found to have an extremely low surface area of 3.3 m^2^ g^−1^. For comparison, the surface area and pore volume of *f*-OMC, FeS@*f*-OMC composite, and pristine FeS are listed in Table 1.

### 2.2. Electrochemical Properties in the Presence of Na/Na^+^ during Charge/Discharge

**Figure 6:**
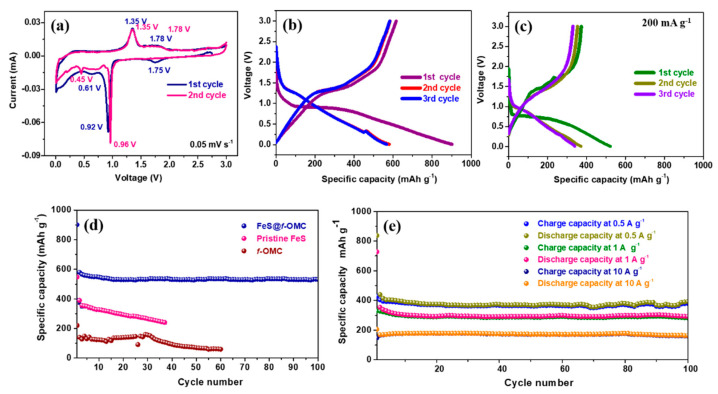
Electrochemical properties of the FeS@*f*-OMC composite: (**a**) Cyclic voltammetry curves, charge/discharge profiles of (**b**) FeS@*f*-OMC, (**c**) pristine FeS, cycle performances at (**d**) 200 mA g^−1^, and (**e**) current densities of 0.5, 1, and 10 A g^−1^.

The electrochemical properties of the synthesized FeS@*f*-OMC composite vs. Na/Na^+^ were analyzed in detail via a series of electrochemical tests carried out at room temperature. Figure 6a shows the cyclic voltammograms of the composite, obtained from 0.01 to 3.0 V. In the 1st cycle, a small cathodic peak is observed at 1.75 V, which indicates the partial sodiation of FeS, which results in the formation of a Na*_x_*FeS phase [34]. The sharp peak at 0.92 V and broad peak at 0.61 V denote the complete reduction of FeS, according to the conversion reaction, forming Fe and Na_2_S, along with Na-rich phases, depending on the extent of Na-ion transfer [15,35]. The sodiation process in the initial cycle also leads to the irreversible formation of a solid electrolyte interphase (SEI) [35]. Further, the sharp peak at 1.35 V during the anodic scan indicates the desodiation of Na_2_S (to form Na_2_FeS_2_), whereas the broad peak at 1.78 V can be attributed to extended desodiation, which leads to the formation of the Na_2−*x*_FeS_2_ species [35,36]. In the second cycle, unlike the initial sodiation process, the reduction peak appears at 0.96 V, denoting the transformation of Na_2−*x*_FeS_2_ to Na_2_FeS_2_. In addition, the lower reduction peak sharpened in the second cycle and showed a minor shift. This could be a result of the reduction of the Na_2_FeS_2_ phase confined in the 2D mesoporous channels, leading to the formation of Na_2_S and Fe. The oxidation peaks overlap entirely in the subsequent anodic scan, indicating the excellent reversibility of the FeS@*f*-OMC composite electrode [37].

Galvanostatic charge/discharge tests were initially carried out at a low current density of 200 mA g^−1^. The FeS@*f*-OMC composite exhibited a well-defined plateau in the initial sodiation process at approximately 0.97 V, followed by a sloping region until deep discharge (0.01 V), leading to the successive formation of the Na*_x_*FeS_2_ and Fe + Na_2_S phases. In addition, a high initial discharge capacity of 902 mAh g^−1^ was obtained, suggesting the complete sodiation of the FeS@*f*-OMC composite, along with the irreversible formation of the SEI layer (Figure 6b). The composite anode exhibited a charge capacity of 615 mAh g^−1^, corresponding to a Coulombic efficiency (CE) of 68% in the initial cycle. Such a low initial CE has been observed previously as well, in the case of conversion-based electrode materials, and can be correlated to the irreversible loss of Na^+^ during SEI layer formation in the initial cycle and the sluggish conversion reaction process [35,36]. The electrode exhibited a stable capacity of 520 mAh g^−1^ in the second cycle, with a sloping plateau region completely different from that of the initial cycle, consistent with previous reports [35,36]. During the initial desodiation process, the Na*_x_*FeS_2_ species are generated reversibly and form Na_2_S + Fe upon subsequent desodiation. This change in the reaction pathway is manifested in the voltage profile of the FeS@*f*-OMC composite and the pristine FeS from the second cycle onwards. Furthermore, in the case of the FeS@*f*-OMC composite, the voltage plateaus from the second cycle onwards overlap, implying good reversibility of the electrochemical reaction. In contrast, the pristine FeS did not show good reversibility (Figure 6c). In addition, the discharge/charge capacity of the pristine FeS was lower than that of the FeS@*f*-OMC composite. To determine the capacity contribution of the *f*-OMC encapsulating FeS, galvanostatic charge/discharge studies were also conducted with the *f*-OMC electrode. Figure 6d shows the comparative cycling performances of the FeS@*f*-OMC, pristine FeS, and *f*-OMC electrodes. The pristine FeS electrode exhibited a gradual capacity decay as the cycling progressed, whereas the FeS@*f*-OMC composite displayed a stable cycle performance for 100 cycles. The stable capacity retention observed in the FeS@*f*-OMC cells can be attributed to the presence of FeS nanoparticles confined within the parallel channels of the OMC matrix. This leads to efficient Na-ion transport and enhances the reversibility of the sodiation/desodiation process. The improved capacity of the FeS@*f*-OMC composite could be attributed to reversible Na-ion storage in *f*-OMC at low voltages. Furthermore, the cycle performance of the FeS@*f*-OMC composite at various current densities was evaluated for 100 cycles. At current densities of 0.5, 1, and 10 A g^−1^, the FeS@*f*-OMC composite showed stable cycle performance, yielding capacity retentions of 379, 283, and 165 mAh g^−1^ after 100 consecutive sodiation/desodiation cycles (Figure 6e).

**Figure 7:**
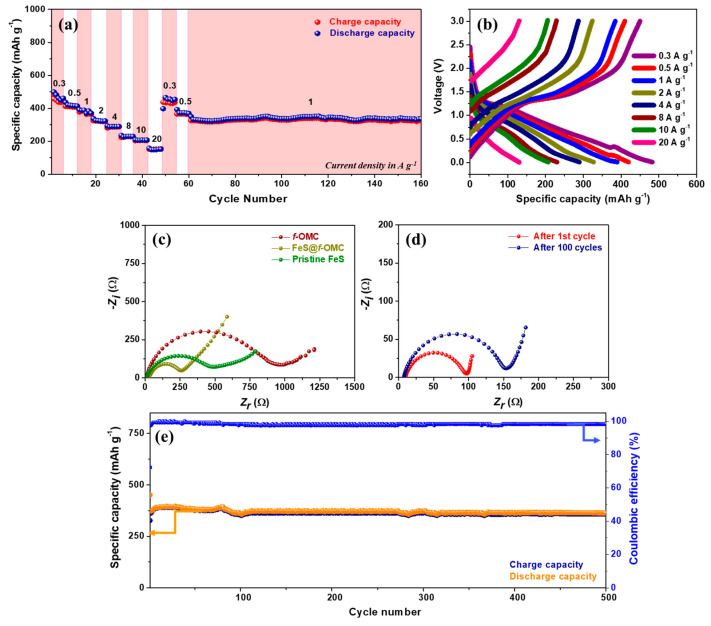
(**a**) Rate performance along with (**b**) corresponding charge/discharge profiles of the FeS@*f*-OMC composite, EIS spectra of cells of (**c**) pristine FeS, *f*-OMC, and FeS@*f*-OMC, as well as that of (**d**) an FeS@*f*-OMC cell after cycling and (**e**) cycle performance at 2 A g^−1^.

OMCs generally display good rate performance, owing to their high-surface-area induced wettability and the presence of sufficient electrolyte percolation pathways. Therefore, the high-rate performance of the FeS@*f*-OMC composite was evaluated by increasing the current density from 0.3 A g^−1^ in a stepped fashion (Figure 7a). Interestingly, the composite retained capacities of 483, 419, 389, 327, 289, 230, 207, and 133 mAh g^−1^ at current densities of 0.3, 0.5, 1, 2, 4, 8, 10, and 20 A g^−1^, respectively. Such high-rate performances are rarely reported for SIB anode materials. For example, typical anode materials, such as hard carbon and MXenes, deliver much lower capacities, even at lower current densities, than those used in the FeS@*f*-OMC composite [38,39,40,41]. Further, after switching back to the low current density of 0.3 A g^−1^, the initial discharge capacity was retained. Subsequently, after the high-rate performance tests, the FeS@*f*-OMC composite exhibited stable cycle performance at 1 A g^−1^.

The electrochemical impedance spectroscopy (EIS) spectra of the FeS@*f*-OMC composite, *f*-OMC, and pristine FeS were analyzed before and after cycling (Figure 7c,d). The Nyquist plots of all cells contains a semicircle in the high- to low-frequency region, and we calculated the *x*-axis intercept of the straight line in the low-frequency region (Warburg element) related to the diffusion processes to determine the charge transfer resistance (*R_ct_*). The fresh cells of pristine OMC showed the highest *R_ct_*, followed by the pristine FeS and FeS@*f*-OMC composites. However, as shown in Figure 7d, the *R*_ct_ of the FeS@*f*-OMC cells decreased after the 1st cycle, due to the formation of ultrafine nanocrystals via the conversion reaction of FeS and the simultaneous formation of a stable SEI layer in the initial cycle [11,33]. After 100 cycles, the *R_ct_* of the cells increased slowly, indicating the gradual growth of the SEI layer, with continuous cycling. Long-term cycling studies of the composite carried out at 2 A g^−1^ also revealed excellent stability and reversibility for 500 sodiation/desodiation cycles, retaining almost 100% CE (Figure 7e). This promising high-rate performance of the FeS@*f*-OMC composite can be attributed to the FeS nanoparticles confined within the 2D parallel mesoporous channels of the OMC framework, which provide short Na-ion diffusion pathways during cycling. In addition, the mesoporous structure and N-heteroatom doping enhanced the wettability of the electrolyte and improved electrolyte percolation in the FeS@*f*-OMC electrode. These characteristics, accompanied by the improved contact area with the active material, facilitate the rapid transfer of Na^−^ions during the charge/discharge process.

**Figure 8:**
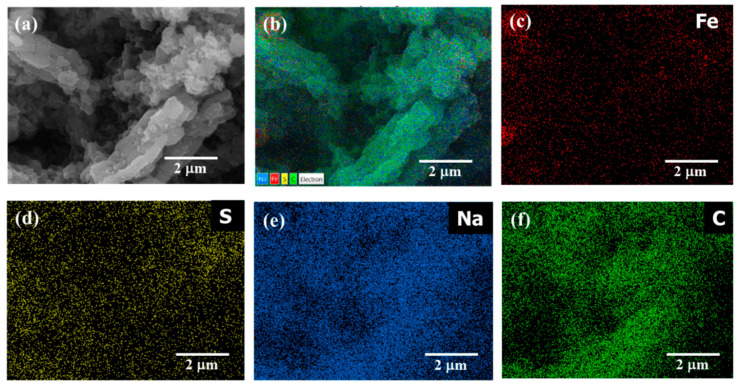
(**a**) FE-SEM image of FeS@*f*-OMC after cycling process, (**b**) HAADF image and the EDS mapping of elements (**c**) Fe, (**d**) S, (**e**) Na, and (**f**) C in the cycled sample.

The morphological analysis of the FeS@*f*-OMC composite recorded after the 160th charge is shown in Figure 8. The FE-SEM image in Figure 8a shows the FeS@*f*-OMC structure, along with polyvinylidene fluoride (PVDF) and conductive carbon. Notably, the structure of the *f*-OMC remained unchanged, even after the high-rate cycling process. The overlay of the high-angle annular dark field (HAADF) image (Figure 8b) and EDS map (Figure 8c–f) reveals the even distribution of Fe, S, Na, and C in the cycled samples, indicating the formation of Na-rich phases of FeS upon sodiation (charging), in agreement with previous reports [10,14,33].

## 3. Materials and Methods

### 3.1. Chemicals and Materials

Poly(ethylene glycol)-*block*-poly(propylene glycol)-*block*-poly-(ethylene glycol) (Pluronic P123, average *M*_w_ ≈ 5800, Aldrich), tetraethylorthosilicate (TEOS, 98%, Aldrich), nitric acid (60%, Samchun Pure Chem. Co., Ltd., Seoul, Korea), sulfuric acid (35.0–30.0%, Samchun Pure Chem. Co., Ltd.), hydrochloric acid (HCl, 35–37%, Samchun Pure Chem. Co., Ltd.), sucrose (Samchun Pure Chem. Co., Ltd.), iron chloride tetrahydrate (FeCl_2_∙4H_2_O, 99.8%, Samchun Pure Chem. Co., Ltd.), thiourea (TU, CH_4_N_2_S, 99% Alfa Aesar), and *N*-methyl pyrrolidone (NMP, 99.5%, Samchun Pure Chem. Co., Ltd.) were used, as received.

### 3.2. Synthesis of FeS@f-OMC Composite

The OMC, having parallel channels, was synthesized by modifying one of our previously reported methods [26]. The synthesis of the OMC is described in detail in the SI. The synthesized OMCs were subjected to acid treatment to facilitate oxidation and functionalization with carboxylic, carbonyl, or hydroxyl groups to achieve dispersion in aqueous solutions. In a typical process, 1 g of OMC was added to a mixture of H_2_SO_4_ and HNO_3_ (1:1, *v*/*v*) and heated to 80 °C for 4 h with constant stirring. The obtained powder sample was washed thoroughly with copious water and dried at 80 °C to obtain acid-functionalized OMC (*f*-OMC). To synthesize the FeS-embedded OMC composite, a simple and scalable aqueous route was chosen. Initially, *f*-OMC was evenly dispersed in a mixed solution of DI water and ethanol (1:1, *v*/*v*) via sonication. Then, FeCl_2_∙4H_2_O (1 g) and thiourea (1 g) were added sequentially to the resultant dispersion, under constant stirring at 80 °C. The mixture was stirred thoroughly to ensure the impregnation of the precursors into the OMC. After the complete evaporation of the solvent, a black powder was obtained. Later, it was ground thoroughly using a mortar and pestle, before heat treatment at 600 °C for 4 h in an argon atmosphere. The FeS-embedded OMC composite (i.e., FeS@*f*-OMC) was obtained after natural cooling.

Pristine FeS was synthesized using a similar procedure, without the addition of the OMCs. The final powder sample was green in color (Figure S1).

### 3.3. Material Characterization

The morphologies of the pristine OMC and FeS@*f*-OMC composite were imaged and assessed using FE-SEM (Philips XL30S FEG) and TEM (TF30ST-300 kV). The crystallinity of the composite was studied using XRD (D2 Phaser Bruker AXS) with a Cu *K*_α_ X-ray source. The chemical states of the elements in the composite were analyzed using XPS (ESCALAB250 VG Scientific) with a monochromatic Al *K*_α_ (1486.6 eV) X-ray source. The thermal stability and content of the active material in the composite was estimated by thermogravimetric analysis (Q50 TA) at a heating rate of 10 °C min^−1^, from room temperature to 800 °C under an air atmosphere. The surface areas and pore size distributions of the samples were analyzed by N_2_ adsorption–desorption isotherms using the Brunauer–Emmett–Teller (BET) method (ASAP 2000).

### 3.4. Electrochemical Characterization

The FeS@*f*-OMC composite was thoroughly mixed with PVDF and super-P in an NMP solution, in an 8:1:1 ratio. The slurry was cast on aluminum foil and allowed to dry overnight at 80 °C. Circular electrodes having a diameter of 1 cm were punched out and directly used as anodes for this study. The cell assembly was achieved using Swagelok^®^ cells in an argon-filled glove box, in which the oxygen and moisture contents were maintained below 1 ppm. Freshly cut sodium metal was used as the reference electrode. The electrolyte consisted of 1 M sodium triflate (NaCF_3_SO_3_) dissolved in diethylene glycol dimethyl ether (DEGDME). A glass fiber membrane was used as the separator. The electrochemical properties of the FeS@*f*-OMC anode were evaluated using an electrochemical workstation (WBCS 3000, WonA Tech Co., Seoul, Korea). Cyclic voltammetry analysis was conducted at a scan rate of 0.05 mV s^−1^, between 10 mV and 3.0 V. Galvanostatic charge/discharge studies were carried out at various current densities to evaluate the electrochemical performance of the composite. The EIS measurements of the cells were carried out using an IM6 impedance analyzer with an amplitude of 5 mV from 100 to 2 MHz.

## 4. Conclusions

We have described a facile and straightforward method for synthesizing FeS-loaded ordered mesoporous carbons via a simple synthetic route. The synthesized FeS@*f*-OMC composite exhibited a high discharge capacity of 520 mAh g^−1^ at a low current density of 200 mA h g^−1^, even after 100 cycles. The presence of ordered mesoporous channels within the carbon matrix facilitates good interfacial contact between the active materials and promotes gradual and effective electrolyte percolation within the electrode, thus favoring fast Na-ion uptake. Accordingly, the FeS@*f*-OMC composite delivered stable cycle performance, retaining a capacity of 363 mAh g^−1^ after 500 cycles at 2 A g^−1^ and a high-rate capability of 133 mAh g^−1^ at a high current density of 20 A g^−1^, indicating excellent prospects for stable and high-rate Na-ion storage applications.

## Figures and Tables

**Table 1:** Surface area and pore volume of *f*-OMC, FeS@*f*-OMC composite, and pristine FeS.

Sample	Surface Area (m^2^ g^−1^)	Pore Volume (cm^3^ g^−1^)
*f*-OMC	1505	1.92
FeS@*f*-OMC	360	0.44
Pristine FeS	3.3	0.01

## Data Availability

Most of the data used during the preparation of the manuscript are included in the Results and Discussion sections.

## Supplementary Materials

The following are available online. Figure S1: As-synthesized pristine FeS and FeS@*f*-OMC anode materials; Figure S2: TEM image of FeS@*f*-OMC composite; Figure S3: FE-SEM images of pristine FeS particles; Figure S4: XRD of FeS@*f*-OMC composite after TGA in air atmosphere; Figure S5: TGA curves in air atmosphere; Section S1: Synthesis of SBA-15 template and CMK-3 Ordered Mesoporous Carbon; Section S2 Estimation of FeS content in FeS@*f*-OMC composite by TGA.
