# Supplementary material for: A Hierarchically Ordered Mesoporous-Carbon-Supported Iron Sulfide Anode for High-Rate Na-Ion Storage
Source: Molecules. 2021 Jul 18;26(14):4349. doi: 10.3390/molecules26144349 (PMC8307886; doi:10.3390/molecules26144349)
Supplement: Supplementary file 1 [file molecules-26-04349-s001.zip › molecules-1290022-supplementary.pdf]

Supporting Information

A Hierarchically Ordered Mesoporous-Carbon-Supported Iron Sulfide Anode for High-Rate Na-ion Storage

Anupriya K. Haridas [1], Natarajan Angulakshmi [1], Arul Manuel Stephan [2], Younki Lee [1,]* and Jou-Hyeon Ahn [1,]*

[1] Department of Materials Engineering and Convergence Technology, Gyeongsang National University, 501 Jinju-daero, Jinju 52828, Korea
[2] Electrochemical Power Sources Division, CSIR – Central Electrochemical Research Institute, Karaikudi 630 006, India
Correspondence: ylee@gnu.ac.kr (Y.L.); jhahn@gnu.ac.kr (J.H.A.)

List of content:

- **Section S1** Synthesis of SBA-15 template and CMK-3 Ordered Mesoporous Carbon
- **Section S2** Estimation of FeS content in FeS@*f*-OMC composite by TGA
- **Figure S1** As-synthesized pristine FeS and FeS@*f*-OMC anode materials
- **Figure S2** TEM image of FeS@*f*-OMC composite
- **Figure S3** FE-SEM images of pristine FeS particles
- **Figure S4** XRD of FeS@*f*-OMC composite after TGA in air atmosphere
- **Figure S5** TGA curves in air atmosphere

Section S1 Synthesis of SBA-15 template and CMK-3 Ordered Mesoporous Carbon

Initially, the *CMK-3 Ordered Mesoporous Carbon* derived from the SBA-15 silica template was synthesized by modifying our previous work utilizing P123 as a surfactant, TEOS as silica source and sucrose as carbon source. Briefly, 2.0 g of P123 was dissolved in 2 M HCl (60 mL) at 40 °C with continuous stirring. 4.2 g TEOS was then added to the solution and stirred vigorously to ensure homogeneous mixing. The mixture was kept at 40 °C for 24 h and later aged in a Teflon jar at 130 °C for 3 consecutive days. The product was collected by vacuum filtration and dried overnight at 80 °C. The calcination of the obtained white powder at 550 °C for 4 h in air atmosphere yielded the ordered mesoporous SBA-15 silica template. To prepare the CMK-3 ordered mesoporous carbon, 2.0 g of sucrose was SBA-15 via an incipient wetness impregnation method. Afterward, the sample was dried at 80 °C for 12 h. This impregnation/drying step was carried out multiple times to ensure the complete filling of the mesopores in the SBA-15 template. Finally, the carbonization of the sucrose impregnated SBA-15 was carried out by heat treatment at 850 °C for 4 h at a ramp rate of 5 °C min^{-1}. The obtained black colored powder was soaked in 7 wt% HF solutions for 36 h to remove the silica template altogether. The final product was washed thoroughly with a copious amount of DI water and dried at 80 °C to obtain the CMK-3 ordered mesoporous carbon.

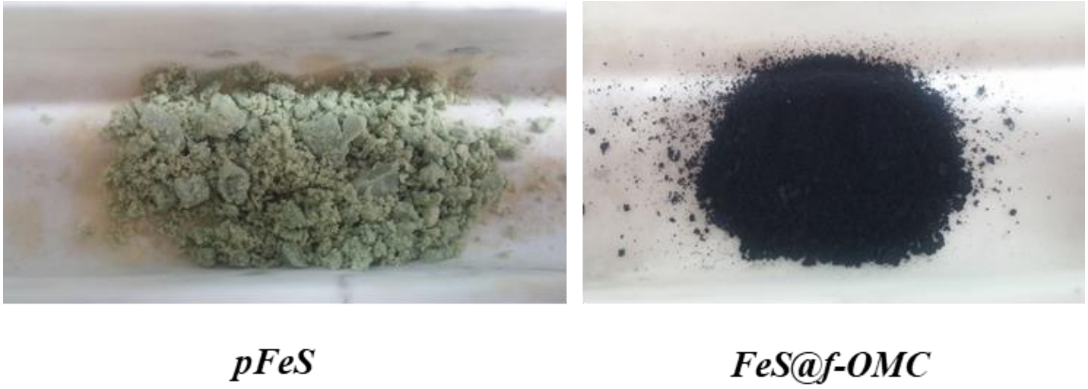

Figure S1 As-synthesized pristine FeS and FeS@*f*-OMC anode materials.

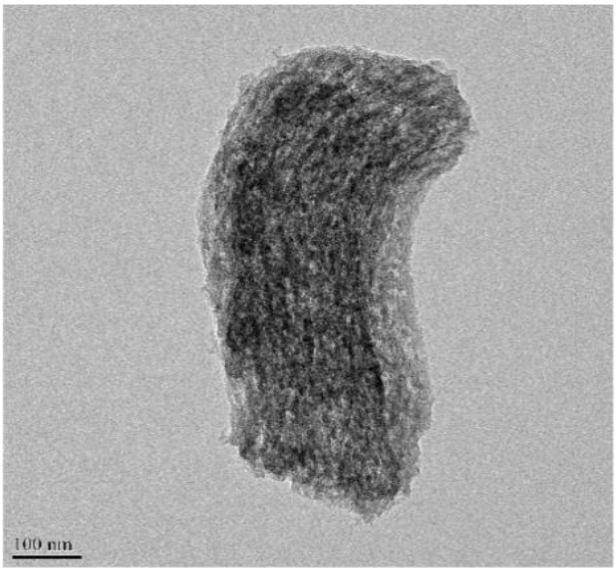

Figure S2 TEM image of FeS@*f*-OMC composite.

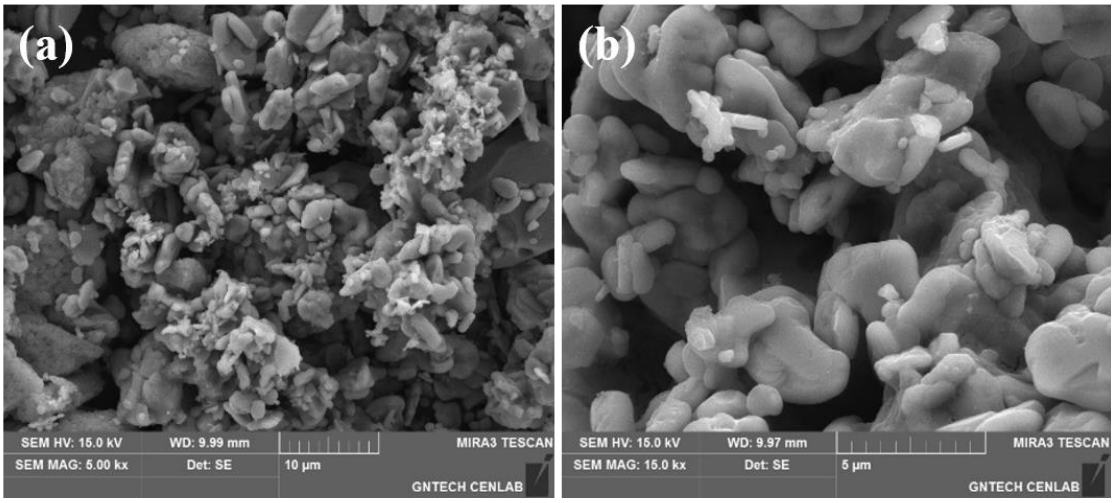

Figure S3 FE-SEM images of pristine FeS particles.

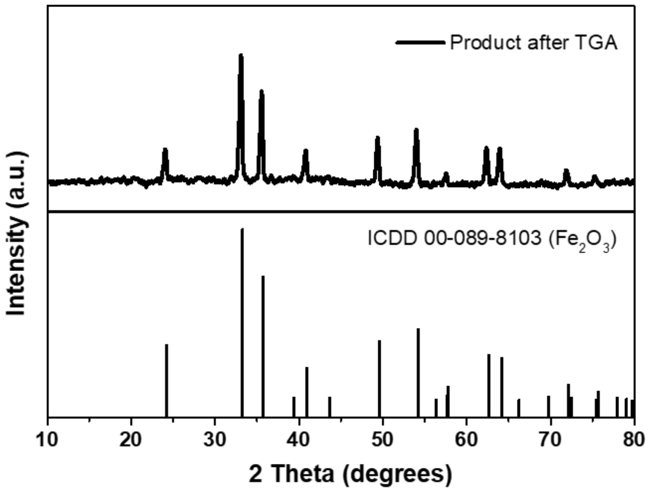

Figure S4 XRD of FeS@*f*-OMC composite after TGA in air atmosphere.

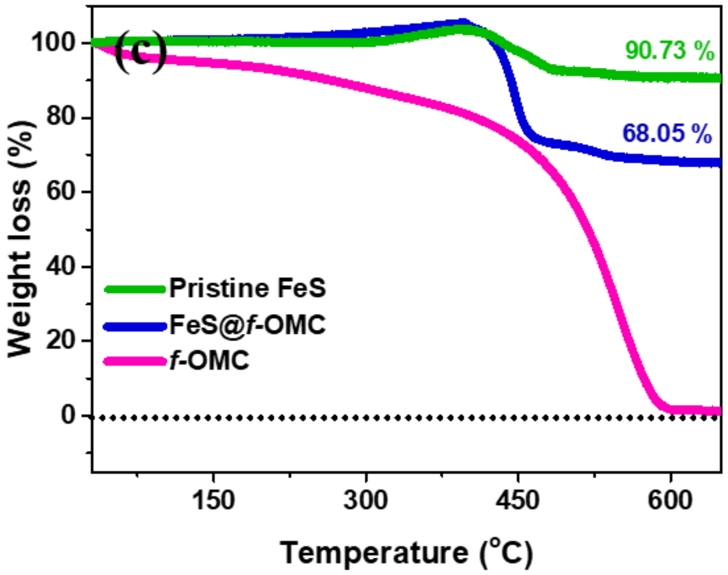

Figure S5 TGA curves in air atmosphere.

Section S2 Estimation of FeS content in FeS@*f*-OMC composite by TGA

$$\%C = \frac{R1 - R2}{0.90824 - R2}$$

where %C denotes the FeS content in the composite, R1 the mass residue of FeS@*f*-OMC composite (from TGA curve, blue line), R2 the mass residue of *f*-OMC (from TGA curve, pink line), and 0.90824 the theoretical mass residue of pristine FeS assuming final product after heat treatment in air as Fe_2O_3. Accordingly, R1 = 68.05 %, R2 = 0% (from TGA)

FeS content = 75% ; *f*-OMC content = 15%.
